# Internal Marketing and Service‐Oriented Organizational Citizenship Behavior Among Medical and Nursing Staff in Nursing Homes

**DOI:** 10.1155/jonm/1415183

**Published:** 2026-11-15

**Authors:** An-Jin Shie, Junhui Liu, Jingxian Xie, En-Min Xu

**Affiliations:** ^1^ College of Business Administration Huaqiao University Quanzhou China hqu.edu.cn; ^2^ International College Krirk University Bangkok Thailand krirk.ac.th; ^3^ International College Huaqiao University Quanzhou China hqu.edu.cn; ^4^ School of Information Management Nanjing University Nanjing China nju.edu.cn

**Keywords:** care-giving workload, elderly care, emotional labor, internal marketing, nursing home medical and nursing staff, nursing homes, response surface methodology, service-oriented organizational citizenship behavior

## Abstract

**Introduction:**

This study examines the association between internal marketing and service‐oriented organizational citizenship behavior among medical and nursing staff in eldercare institutions and investigates the mediating roles of emotional labor and care‐giving workload.

**Design:**

A three‐wave time‐lagged survey design.

**Methods:**

This study included 515 medical and nursing staff from 14 nursing homes in Beijing. Nursing homes were initially selected through random sampling, and data were collected using online self‐report questionnaires. Structural equation modeling and response surface methodology were used to test the hypotheses and examine interaction effects.

**Results:**

Internal marketing had a significant positive effect on service‐oriented organizational citizenship behavior among medical and nursing staff in nursing homes. Significant indirect effects were observed through both dimensions of emotional labor (surface acting and deep acting). In contrast, the indirect effect through care‐giving workload was not significant, although workload was negatively associated with service‐oriented organizational citizenship behavior. Response surface analysis further revealed a significant curvilinear pattern along the line of incongruence between internal marketing and care‐giving workload. Further response surface analysis indicated a nonlinear joint effect of internal marketing and care‐giving workload on service‐oriented organizational citizenship behavior, with an inverted U‐shaped pattern along the line of incongruence.

**Conclusion:**

This study broadens the understanding of service‐oriented organizational citizenship behavior among medical and nursing staff in nursing homes by considering employees’ emotional regulation and work demands alongside internal marketing. By distinguishing the indirect associations involving emotional labor from the nonlinear joint relationship involving care‐giving workload, it offers a more differentiated perspective on the association between organizational support and discretionary service behavior.

**Implication for Nursing Management:**

Nursing home administrators and nursing managers should strengthen internal marketing through effective communication, staff recognition, and professional development opportunities. Emotional support and emotion‐regulation training may help medical and nursing staff manage care‐related emotional demands. Regular workload assessments, appropriate staffing, and reasonable task allocation may further help managers tailor internal marketing practices to employees’ work demands and foster service‐oriented organizational citizenship behavior.

## 1. Introduction

Against the backdrop of accelerating global population aging, advances in communication media and rising economic standards have increased older adults’ awareness of and expectations for health and care services [1], driving continued growth in demand for high‐quality, personalized eldercare. However, the eldercare sector has long faced structural challenges, including high staff turnover, intense workloads, and weak professional identity [2]. A common and concerning situation has followed: Many medical and nursing staff in nursing homes tend to follow prescribed procedures and complete only basic care tasks, while showing reluctance toward service‐oriented organizational citizenship behavior (SO‐OCB). Such behavior involves voluntary actions to meet older adults’ expectations for high‐quality care, including attending to residents’ psychological states, helping colleagues beyond assigned duties, and proactively informing families of subtle changes in residents’ daily lives. Previous research indicates that employees who engage in SO‐OCB can respond more precisely to clients’ individualized needs, thereby improving satisfaction and strengthening organizational competitiveness over time [3]. Older residents also have needs for emotional companionship and psychological support [4]. By showing concern for residents’ psychological states, listening patiently to their emotional needs, and communicating promptly with families beyond their basic duties, staff who engage in SO‐OCB can turn procedural service into compassionate care [5]. These actions can substantially improve residents’ quality of life and subjective well‐being, strengthen families’ trust in and satisfaction with nursing homes, and, in an increasingly competitive eldercare market, increase institutional occupancy rates and overall competitiveness. Therefore, how to encourage and sustain SO‐OCB among medical and nursing staff in nursing homes has become an important question.

As a management approach that attends to employees’ needs, internal marketing (IM) offers a useful perspective on this issue. IM treats employees as internal customers and seeks to improve their working conditions through managerial support, training, communication, and empowerment. It may also encourage employees to contribute to service beyond their prescribed duties [6–8]. Internal exchanges between organizations and employees can help employees develop service relationships oriented toward service recipients’ needs [9–11]. Empirical research has found that IM is positively associated with general organizational citizenship behavior and that job satisfaction partially explains this relationship [12]. IM has also been positively associated with employees’ organizational citizenship behavior and customer orientation [13]. In the hotel industry, Chang [14] further found a positive association between IM and employees’ SO‐OCB, with employees’ psychological attachment to the organization playing an indirect role. These studies provide a basis for examining the relationship between IM and SO‐OCB among medical and nursing staff in nursing homes. Their SO‐OCB reflects both the proactive contributions emphasized in organizational citizenship behavior and the attention to service recipients’ needs emphasized in customer orientation. It also shares the service‐oriented character of SO‐OCB among hotel employees [15, 16]. However, in eldercare, this behavior is embedded in professional care and long‐term service relationships and involves sustained responses to older adults’ complex care needs. Its expression and the conditions under which it occurs may therefore be distinctive, and the applicability of previous findings to this group remains to be tested. Whether IM can promote SO‐OCB among medical and nursing staff in nursing homes, and through what mechanisms, remains unclear.

Ongoing care interactions require medical and nursing staff in nursing homes to respond to older residents’ emotional and individual needs while regulating their own feelings and expressions [17, 18]. This emotional labor (EL) is shaped by the emotional demands of care, as well as by organizational support and opportunities for autonomy [19]. Through managerial support, training, and empowerment, IM may provide staff with the resources and discretion needed to navigate complex care interactions, thereby shaping their selection and enactment of EL strategies [20]. From a conservation of resources (COR) theory perspective, these organizational resources may ease the strain associated with sustained emotional demands and support staff in regulating their emotions [21, 22]. Different EL strategies may entail different levels of resource expenditure, affecting whether staff can engage in additional voluntary service after completing their required care tasks [23, 24]. From a self‐determination theory (SDT) perspective, support for competence and autonomy may help staff identify with care goals and the associated emotional demands, shaping their autonomous motivation to regulate their emotions [25, 26]. This motivation may, in turn, influence their willingness to continue engaging in SO‐OCB beyond their prescribed duties [26, 27]. Existing research provides preliminary evidence for these pathways. Altaş et al. [28] found that supervisor support and work autonomy were associated with employees’ EL strategies, while Ma [29] found an association between these strategies and SO‐OCB. IM may therefore be linked to SO‐OCB through staff’s EL strategies and the resource and motivational conditions associated with them. This mediating pathway remains to be tested.

Alongside regulating their emotions and responding to older residents’ needs, medical and nursing staff in nursing homes must carry out routine tasks such as assisting with eating, cleaning, and health monitoring. Continuing care demands are thus an important source of workload [30, 31]. How demanding staff perceive this work to be is also related to the support available while they perform it. Among nurses, van den Oetelaar et al. [32] found that coworker support, as well as the time required for care, was associated with perceived workload. Training, managerial support, and empowerment provided through IM may likewise improve working conditions by giving staff the skills, resources, and discretion needed to manage care demands [20]. From a COR perspective, access to resources may ease the strain staff experience when care demands exceed their capacity to cope, potentially reducing perceived workload [33, 34]. Perceived workload may, in turn, be relevant to their capacity for service beyond formal duties. When routine tasks continually consume time and energy, staff may prioritize required care over voluntary contributions such as helping colleagues and improving services [31, 35]. Limited discretion over how to organize work, combined with difficulty meeting increasing care demands, may also frustrate the needs for autonomy and competence and weaken motivation for discretionary service, consistent with SDT [27, 36]. We therefore propose that IM may be associated with greater SO‐OCB through lower perceived care‐giving workload (CW), leaving staff with more resources and willingness to engage proactively in service after completing their required tasks.

Based on the above analysis, this study addresses two research questions: (1) Can IM promote SO‐OCB among medical and nursing staff in nursing homes? (2) Does IM influence their SO‐OCB through two mediating pathways, EL and CW?

To answer these questions, this study focuses on frontline medical and nursing staff in nursing homes. Drawing on COR and SDT, it develops a dual‐mediator model with EL and CW as mediators. By examining the relationships between IM and employees’ emotion regulation, perceptions of CW, and engagement in service beyond their prescribed duties, the study aims to reveal the mechanisms linking IM to SO‐OCB from the perspectives of resources and motivation. The abbreviations used in this study are listed in Table A1 in the Appendix.

## 2. Theoretical Model and Hypotheses

### 2.1. Direct Effect of IM on SO‐OCB

Boukis et al. [8] define IM as a strategic approach that integrates marketing principles with human resource management to motivate employees across organizational levels. Effective IM aligns employee needs with organizational goals, thereby enhancing perceived service quality. By providing managerial support, autonomy, and individualized attention, IM promotes resource optimization, teamwork, and customer satisfaction [37]. In eldercare institutions management, IM serves as a practical tool for optimizing eldercare workforce management. Through observation and communication, managers can better understand employee needs and develop supportive initiatives by treating staff as “internal customers” [38].

According to COR theory, employees cope with job demands by acquiring resources to minimize depletion and maintain work performance [39]. In the eldercare context, resource scarcity—such as limited emotional support and autonomy—contributes to stress and behavioral decline [40]. IM can address these deficits by providing managerial support, granting autonomy, and attending to employees’ needs [28]. Prior studies have confirmed that IM fosters job engagement [41], enhances service interactions and satisfaction [42], promotes collaboration and well‐being [43], and increases SO‐OCB and organizational commitment [44, 45]. Based on the above analysis, the following hypothesis is proposed:

• H1: Internal marketing has a positive effect on SO‐OCB.

### 2.2. The Mediating Role of EL

EL plays a central role in interactions between eldercare workers and residents, yet its definition and structure remain debated. EL is commonly viewed as a multidimensional construct, comprising surface acting emotional labor (SAEL) and deep acting emotional labor (DAEL) [46]. DAEL involves aligning internal feelings with organizational expectations to express genuine emotions, while SAEL entails altering outward expressions without changing inner feelings—often used as a coping mechanism [47].

In eldercare institutions, where emotional demands are high [48], DAEL is associated with positive psychological outcomes and stronger client engagement, whereas prolonged SAEL use may lead to exhaustion and lower job satisfaction [49]. According to COR theory, organizational resources reduce stress and promote DAEL strategies [50]. IM practices—such as managerial support, autonomy, and responsiveness—can foster psychological safety, encourage DAEL, and buffer the adverse effects of SAEL [51]. Based on the above analysis, the following hypotheses are proposed:

• H2a: Internal marketing has a negative effect on SAEL.
• H2b: Internal marketing has a positive effect on DAEL.

SDT posits that individuals are intrinsically motivated when their basic psychological needs for autonomy, competence, and relatedness are fulfilled [52]. In eldercare settings, however, high work pressure and frequent EL often require caregivers to regulate their emotions during service interactions. Employees typically adopt either SAEL or DAEL strategies [53]. SAEL involves superficial expression adjustments, while DAEL entails internal emotional alignment with job expectations. Prolonged use of SAEL can erode autonomy and competence, leading to emotional exhaustion and reduced SO‐OCB [53]. In contrast, DAEL fosters role identification and encourages voluntary, high‐quality service behavior [54]. Based on these insights, the following hypotheses are proposed:

• H3a: SAEL has a negative effect on SO‐OCB.
• H3b: DAEL has a positive effect on SO‐OCB.

COR theory suggests that individuals are motivated to acquire and protect resources to cope with stress, and resource loss leads to burnout and reduced performance [50, 55]. In eldercare, prolonged EL often depletes emotional resources, increasing the risk of exhaustion [53]. IM, as a form of organizational support, provides both material and psychological resources that help caregiving staff manage emotional strain [56]. While DAEL promotes enthusiasm and SO‐OCB, SAEL is linked to exhaustion and reduced motivation [47]. IM can thus indirectly enhance SO‐OCB by fostering DAEL and reducing reliance on SAEL [51]. Based on the above analysis, the following hypothesis is proposed:

• H4: Emotional labor mediates the relationship between internal marketing and SO‐OCB.

### 2.3. The Mediating Role of CW

CW is an important consideration for staff performance and care quality in eldercare. High occupational stress and workload have been associated with greater absenteeism, poorer care quality, and increased risks to patient safety [57]. As populations age, increasing demands on medical and nursing staff in nursing homes may further undermine job satisfaction and willingness to provide service [58, 59]. IM may help nursing homes mitigate these pressures by improving employee satisfaction and loyalty and reducing perceived CW [60]. Heavy CW may also deplete employees’ resources, weakening their motivation to provide service and engage in organizational citizenship behavior. COR proposes that individuals seek to acquire and retain resources to cope with work‐related stress [61]. By providing material and psychological resources through IM, nursing homes may alleviate stress associated with resource shortages and reduce perceived CW [62]. Even when the number of care tasks cannot be reduced in the short term, managerial support may help staff coordinate tasks and obtain assistance with decisions [63]. Training may strengthen their ability to manage complex care tasks [64], while job autonomy allows them to adjust the sequence of tasks and how care is delivered [65]. Attention to employees’ needs and managers’ willingness to listen may also strengthen their sense of support and belonging, easing feelings of isolation when workloads are heavy [66]. Thus, although IM may not directly reduce the number of tasks required to meet older residents’ care needs, timely assistance, task coordination, and autonomy in organizing work may narrow the gap between care demands and available resources, thereby reducing perceived CW [67, 68].

• H5: Internal marketing has a negative effect on perceived care‐giving workload.

In the eldercare sector, CW directly impacts both working conditions and SO‐OCB [58]. Drawing on SDT, prolonged exposure to heavy CW undermines employees’ autonomy, competence, and relatedness—core psychological needs that drive intrinsic motivation and prosocial behavior [42]. Excessive tasks and time pressure restrict autonomy, making it harder for staff to act proactively [69]. Resource depletion reduces perceived competence and increases burnout, weakening willingness to deliver quality care [70]. Additionally, CW can erode relatedness by fostering detachment from clients and colleagues, reducing key dimensions of SO‐OCB such as helping behavior and empathetic care [71]. Based on the above analysis, the following hypothesis is proposed:

• H6: Care‐giving workload has a negative effect on SO‐OCB.

Xanthopoulou et al. [50] suggested that individuals with more resources may respond more proactively to job demands, perceive less workload, and be more willing to provide high‐quality service. IM may provide resources that reduce CW among medical and nursing staff in nursing homes. Lower perceived CW may, in turn, support their autonomy, sense of competence, and sense of belonging, creating conditions for SO‐OCB. CW is therefore proposed as a mediator in this relationship. From a COR perspective, service beyond prescribed duties requires employees to invest additional resources [72]. Interviews with nurses in nursing homes indicate that demanding routine care tasks take up time and require staff to prioritize among residents’ needs [63]. When perceived CW is lower, staff may have more attention and scope to act on older residents’ needs, assist colleagues, or improve services. Research involving nurses has also linked organizational service‐oriented management practices to discretionary service behavior through changes in employees’ work behavior [73]. Taken together, these arguments suggest that CW may mediate the relationship between IM and SO‐OCB among medical and nursing staff in nursing homes. We therefore propose the following hypothesis:

• H7: Care‐giving workload mediates the relationship between internal marketing and SO‐OCB.

In summary, the theoretical model of this study is presented in Figure 1.

**FIGURE 1:**
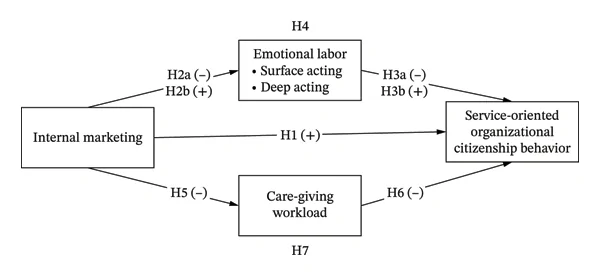
Theoretical model.

## 3. Research Methodology

### 3.1. Sample and Data Collection

Data were collected through a questionnaire survey, with responses screened according to the study’s eligibility and data‐quality criteria. Beijing provides a relevant setting for this research. At the end of 2023, it had 4.948 million older residents, who accounted for 22.6% of its resident population. Its relatively well‐developed eldercare system includes public, private, and mixed‐ownership institutions, allowing staff service behavior to be examined across different types of nursing homes. Beijing has also continued to promote training and incentives for medical and nursing staff in eldercare while emphasizing professional care and responsiveness to older adults’ individual needs. These features make it pertinent to examine whether organizational support is associated with proactive care, assistance, and service improvement among staff. Nursing homes in Beijing were therefore selected as the study setting.

According to the Beijing Report on the Development of Aging Services (2023), 571 nursing homes were operating across Beijing’s 16 districts. Using the institutional list available at the time of the survey, we identified homes that met the inclusion criteria and constructed a district‐stratified sampling frame. To account for both geographic coverage and the distribution of eligible homes, we planned to select one home from each district and an additional home from each of the four districts with the largest numbers of eligible homes. This yielded a target sample of 20 homes: one from each of 12 districts and two from each of the remaining four. Within each district, eligible homes were numbered and selected without replacement using computer‐generated random numbers in accordance with the planned allocation. Following coordination by government departments and a research briefing, 14 of the 20 selected homes agreed to participate, an institutional participation rate of 70%. To assess potential selection differences, we descriptively compared the 14 participating homes with the six nonparticipating homes using information available at the sampling stage. The groups were broadly similar in district location, ownership, and bed capacity, and the scale of eldercare resources in their respective districts showed no marked difference.

Participants were medical and nursing staff employed by the participating nursing homes. Staff who had left or intended to leave their positions were excluded. To reduce the risk of common method bias, we used a three‐wave time‐lagged survey, with 10 working days between consecutive waves. The survey was administered online. Each participating home arranged a group briefing for eligible staff, after which survey links were sent by email or messaging applications. Respondents completed the questionnaires independently on a mobile phone or computer. A unique participant code was used to match their responses across the three waves. In Wave 1, survey invitations were sent to 700 employees to collect demographic information and measure IM. After excluding nonresponses, duplicate submissions, invalid participant codes, and responses with missing key items, 623 valid responses remained. In Wave 2, these 623 employees were invited to complete measures of emotional labor and CW; 561 valid responses were matched to Wave 1 using participant codes. In Wave 3, the 561 matched employees were invited to complete the SO‐OCB measure. The final analytic sample comprised 515 employees with valid matched responses across all three waves. Sample characteristics are reported in Table 1.

**TABLE 1:** Descriptive statistics of sample characteristics.

Item	Category	Frequency	Percentage (%)
Gender	Male	215	41.75
	Female	300	58.25

Age	25 years and below	42	8.16
	26–30 years	40	7.77
	31–35 years	28	5.44
	36–40 years	39	7.57
	Above 41 years	366	71.07

Education	High school or below	178	34.56
	Junior college	271	52.62
	Bachelor’s degree	66	12.82
	Master’s degree or above	0	0.00

Marital status	Unmarried	123	23.88
	Married	392	76.12

### 3.2. Variable Measurement

Study variables were measured using items adapted from established scales and items developed for this study. The initial item pool was tailored to the work context of medical and nursing staff in nursing homes. Items drawn from English‐language scales were translated into Chinese and then back‐translated by a bilingual researcher who had not seen the original items. The research team compared the back‐translations with the originals and resolved differences in meaning. For items drawn from Chinese‐language scales, we checked their original meanings and adapted institutional terms and work‐related wording that did not fit the nursing home setting. For example, the IM item “Do you think the company offers opportunities for ongoing education and training?” was adapted to “Do you think the nursing home provides me with various training opportunities?” to refer to respondents’ own institution and training experiences. Before pilot testing, four experts in eldercare and organizational behavior independently reviewed the initial items for construct relevance, clarity, suitability for the care setting, and content overlap. We calculated the item‐level content validity index (I‐CVI) from their relevance ratings. Using the I‐CVI ≥ 0.78 criterion proposed by Polit et al. [74], we revised or removed items below the threshold after considering the experts’ comments. Twenty eligible care staff then completed a pilot test and commented on item comprehension, similar content, and completion time. We also examined correlations between items within each scale dimension, flagging pairs with correlations above 0.80 for a closer review of possible overlap [75]. Items judged to be substantively redundant were revised or removed with regard to expert feedback and the content coverage of their dimension. Correlations among the retained items were re‐examined in the main sample. Of the 71 initial items, seven were reworded, and 16 were removed, leaving 55 items (see Supporting Material 1 for the questionnaire items). Employees rated each item on a five‐point Likert scale according to how well it reflected their actual work situation, from 1 (“*not at all consistent*”) to 5 (“*very consistent*”).

#### 3.2.1. IM

The IM scale was adapted from the study by Xu et al. [76] to measure perceptions of IM among eldercare service staff. The scale comprises 22 items across two dimensions: managerial support and autonomy, and employee recognition.

#### 3.2.2. EL

EL was measured using the two‐dimensional scale developed by Yang et al. [77], which assesses both SAEL and DAEL. The scale was adapted to fit the working context of nursing homes, allowing for an accurate assessment of EL strategies among eldercare staff and subsequent hypothesis testing. The final version of the scale included eight items.

#### 3.2.3. CW

CW was measured using a 10‐item scale developed for this study. Drawing on Vitale et al. [78] and the nursing home care context, we initially drafted 11 items addressing the physical and psychological strain associated with care work. Following expert review and pilot testing with care staff, a preliminary factor analysis showed that most items clustered on one factor. However, the item “I often have to deal with complaints or criticism from older residents’ family members, which makes me feel stressed.” was associated with a second factor. Because this item primarily captured interpersonal strain arising from interactions with family members rather than demands inherent in care tasks, it was removed after considering both its factor structure and its fit with the CW construct. The remaining 10 items were reassessed using EFA with principal axis factoring. The KMO value was 0.963, and Bartlett’s test of sphericity was significant (*χ* ^2^(45) = 899.605, *p* < 0.001). A single factor was extracted, accounting for 56.142% of the total variance; item loadings ranged from 0.733 to 0.766. These results provide preliminary support for the scale’s single‐factor structure in this sample. Internal consistency reliability and confirmatory factor analysis results are reported in Sections 4.2 and 4.3, respectively.

#### 3.2.4. SO‐OCB

SO‐OCB was measured based on the definition proposed by Guo et al. [79], using the scale developed by Bettencourt and Sheldon [80]. The items were adapted to reflect the work context of medical and nursing staff in nursing homes. The scale consists of 15 items across three dimensions: loyalty, service delivery, and participation, with five items for each dimension.

#### 3.2.5. Demographic Variables

Several demographic variables were included as control variables due to their potential associations with EL and work‐related behaviors. Gender may influence EL tendencies [46]; age is linked to emotional regulation and work cognition [81]; education reflects cognitive capacity and self‐efficacy [82]; and marital status may impact emotional stability via social support [83]. Controlling for these factors reduces confounding effects and enhances the robustness of the findings. Accordingly, gender, age, education level, and marital status were assessed and included in the analysis.

## 4. Results

### 4.1. Common Method Bias Test

Common method bias was assessed using Harman’s single‐factor test and a common latent factor test. In Harman’s test, the first factor accounted for 30.011% of the total variance, below the 40% criterion. Following Podsakoff et al. [84], we then added a common method latent factor with paths to the measurement items in the original five‐factor measurement model. Fit indices for the original model were *χ* ^2^ = 2152.807, df = 1420, TLI = 0.958, CFI = 0.960, RMSEA = 0.032, and SRMR = 0.0349. With the common method factor included, the corresponding values were *χ* ^2^ = 1867.369, df = 1367, TLI = 0.970, CFI = 0.973, RMSEA = 0.027, and SRMR = 0.0371. The mean of the squared standardized loadings on the common method factor was approximately 0.103, below the 0.50 criterion [85]. Together, these results provided no evidence of severe common method bias.

### 4.2. Reliability and Validity Test

Reliability reflects the consistency and stability of measurement results. In this study, internal consistency was assessed using Cronbach’s *α*, with values above 0.80 indicating high reliability, 0.70–0.80 acceptable, and below 0.60 suggesting poor reliability requiring revision. To improve measurement quality, corrected item–total correlation (CITC) was used in the pilot phase to screen items, with those below 0.30 removed to reduce multicollinearity risk.

Reliability analysis, conducted using SPSS, showed that Cronbach’s *α* values for IM, CW, EL, and SO‐OCB all exceeded 0.80, indicating strong internal consistency. All CITC values were above 0.40, further confirming the reliability of the measurement instruments.

Composite reliability (CR) was used to assess internal consistency, while convergent validity was evaluated via average variance extracted (AVE), which reflects the proportion of variance explained by the latent construct. All CR values exceeded 0.70, and AVE exceeded 0.50 for all constructs, indicating acceptable internal consistency and convergent validity (see Table 2).

**TABLE 2:** Reliability and validity testing.

Item	AVE	CR	Cronbach’s *α*
IM	0.598	0.970	0.970
CW	0.561	0.928	0.927
SAEL	0.586	0.850	0.849
DAEL	0.572	0.842	0.841
SO‐OCB	0.519	0.942	0.939

Abbreviations: AVE, average variance extracted; CR, composite reliability; CW, care‐giving workload; DAEL, deep acting emotional labor; IM, internal marketing; SAEL, surface acting emotional labor; SO‐OCB, service‐oriented organizational citizenship behavior.

We assessed discriminant validity by comparing the square root of each construct’s AVE with its construct correlations. As shown in Table 3, each construct’s square root of AVE exceeded its correlations with the other constructs, indicating acceptable discriminant validity.

**TABLE 3:** Pearson correlations and square roots of average variance extracted (AVE).

Item	IM	CW	SAEL	DAEL	SO‐OCB
IM	0.773				
CW	−0.047	**0.749**			
SAEL	−0.283	0.329	**0.766**		
DAEL	0.317	−0.240	−0.277	**0.756**	
SO‐OCB	0.354	−0.286	−0.389	0.475	**0.720**
AVE	0.598	0.561	0.586	0.572	0.519

*Note:* Diagonal values in bold represent the square roots of AVE.

Abbreviations: AVE, average variance extracted; CW, care‐giving workload; DAEL, deep acting emotional labor; IM, internal marketing; SAEL, surface acting emotional labor; SO‐OCB, service‐oriented organizational citizenship behavior.

### 4.3. Confirmatory Factor Analysis

We used CFA to assess whether the relationships between the latent constructs and their observed indicators were consistent with the proposed theoretical structure. Following Møller et al. [86], we evaluated model fit using multiple indices. As shown in Table 4, GFI and NFI were slightly below 0.90 but remained within an acceptable range. The remaining indices met the recommended thresholds.

**TABLE 4:** Model fit indices and overall model evaluation.

Index	CMIN	DF	CMIN/DF	GFI	RMSEA	CFI	NFI	IFI
Recommended	—	—	< 3	> 0.9	< 0.08	> 0.9	> 0.9	> 0.9
Acceptable	—	—	< 5	> 0.8	< 0.10	> 0.8	> 0.8	> 0.8
Observed	2152.807	1420	1.516	0.869	0.032	0.96	0.891	0.96

*Note:* CMIN, minimum discrepancy chi‐square; CMIN/DF, CMIN divided by degrees of freedom.

Abbreviations: CFI, comparative fit index; df, degrees of freedom; GFI, goodness‐of‐fit index; IFI, incremental fit index; NFI, normed fit index; RMSEA, root‐mean‐square error of approximation.

### 4.4. Correlation Analysis

Table 5 presents the correlation coefficients among key variables. Most correlations are significant and align with the hypothesized directions—for example, IM is positively associated with DAEL and SO‐OCB and negatively with SAEL, while CW shows differentiated correlations with SO‐OCB and EL dimensions. The correlation matrix indicates no multicollinearity issues, supporting the use of SEM for further analysis.

**TABLE 5:** Descriptive statistics and correlation analysis of variables.

Item	IM	CW	SAEL	DAEL	SO‐OCB
IM	1.000				
CW	−0.046	1.000			
SAEL	−0.259^∗∗^	0.291^∗∗^	1.000		
DAEL	0.288^∗∗^	−0.212^∗∗^	−0.234^∗∗^	1.000	
SO‐OCB	0.343^∗∗^	−0.272^∗∗^	−0.351^∗∗^	0.427^∗∗^	1.000

Abbreviations: CW, care‐giving workload; DAEL, deep acting emotional labor; IM, internal marketing; SAEL, surface acting emotional labor; SO‐OCB, service‐oriented organizational citizenship behavior.

^∗∗^ *p* < 0.01.

### 4.5. Structural Equation Modeling (SEM)

SEM was employed to assess the fit between the theoretical model and the observed data. As shown in Figure 2 and Table 6, overall model fit was acceptable. GFI and NFI met the acceptable criteria but did not reach the recommended thresholds. These results support the model’s suitability for subsequent path analysis and hypothesis testing.

**FIGURE 2:**
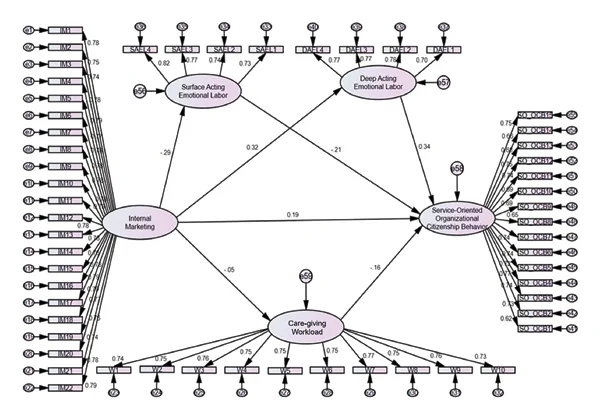
Structural equation modeling.

**TABLE 6:** Model fit indices.

Index	CMIN	DF	CMIN/DF	GFI	RMSEA	CFI	NFI	IFI
Recommended	—	—	< 3	> 0.9	< 0.08	> 0.9	> 0.9	> 0.9
Acceptable	—	—	< 5	> 0.8	< 0.10	> 0.8	> 0.8	> 0.8
Observed	2228.213	1423	1.566	0.864	0.033	0.956	0.887	0.956

*Note:* CMIN, minimum discrepancy chi‐square; CMIN/DF, CMIN divided by degrees of freedom.

Abbreviations: CFI, comparative fit index; df, degrees of freedom; GFI, goodness‐of‐fit index; IFI, incremental fit index; NFI, normed fit index; RMSEA, root‐mean‐square error of approximation.

### 4.6. Path Coefficient Testing and Results

Path analysis within SEM was used to examine the relationships among the independent, mediating, and dependent variables. Path coefficients represent direct relationships between variables, while indirect effects were used to test mediation.

As shown in Table 7, IM was negatively associated with SAEL (*β* = −0.286, C.R. = −5.792, *p* < 0.001, 95% CI [−0.359, −0.231]), supporting H2a, and positively associated with DAEL (*β* = 0.319, C.R. = 6.349, *p* < 0.001, 95% CI [0.215, 0.407]), supporting H2b. The association between IM and CW was not statistically significant (*β* = −0.052, C.R. = −1.118, *p* = 0.264, 95% CI [−0.134, 0.038]); thus, H5 was not supported.

**TABLE 7:** Path coefficients and significance testing results.

Independent variable	Dependent variable	Unstandardized coefficient	Standardized coefficient (*β*)	Standard error (SE)	Bias‐corrected (95%)		*z* (C.R.)	*p*
					Lower bounds	Upper bounds		
IM	SAEL	−0.24	−0.286	0.042	−0.359	−0.213	−5.792	^∗∗∗^
IM	DAEL	0.153	0.319	0.024	0.215	0.407	6.349	^∗∗∗^
IM	CW	−0.04	−0.052	0.036	−0.134	0.038	−1.118	0.264
SAEL	SO‐OCB	−0.12	−0.214	0.026	−0.302	−0.136	−4.559	^∗∗∗^
DAEL	SO‐OCB	0.335	0.34	0.051	0.225	0.448	6.56	^∗∗∗^
CW	SO‐OCB	−0.096	−0.155	0.026	−0.228	−0.084	−3.685	^∗∗∗^
IM	SO‐OCB	0.089	0.188	0.022	0.080	0.281	4.056	^∗∗∗^

Abbreviations: CI, confidence interval; C.R., critical ratio; CW, care‐giving workload; DAEL, deep acting emotional labor; IM, internal marketing; SAEL, surface acting emotional labor; SE, standard error; SO‐OCB, service‐oriented organizational citizenship behavior.

^∗∗∗^ *p* < 0.001.

For the paths to SO‐OCB, SAEL was negatively associated with SO‐OCB (*β* = −0.214, C.R. = −4.559, *p* < 0.001, 95% CI [−0.302, −0.136]), whereas DAEL was positively associated with SO‐OCB (*β* = 0.340, C.R. = 6.560, *p* < 0.001, 95% CI [0.225, 0.448]). These findings supported H3a and H3b, respectively. CW was also negatively associated with SO‐OCB (*β* = −0.155, C.R. = −3.685, *p* < 0.001, 95% CI [−0.228, −0.084]), supporting H6. With these variables included, the direct path from IM to SO‐OCB remained positive and statistically significant (*β* = 0.188, C.R. = 4.056, *p* < 0.001, 95% CI [0.080, 0.281]), supporting H1.

### 4.7. Mediation Analysis

Mediation was assessed using 2000 bootstrap resamples and 95% confidence intervals. Following Preacher and Hayes [87], an indirect, direct, or total effect was considered statistically significant when its bootstrap confidence interval excluded zero. Table 8 presents the results. The indirect effects linking IM to SO‐OCB through SAEL and DAEL were both significant. Together with the significant direct path from IM to SO‐OCB reported in Table 7, these findings were consistent with a statistical pattern of partial mediation, supporting H4. In contrast, the indirect effect through CW was not significant; H7 was therefore not supported. Although the path from IM to CW was not significant, CW was significantly associated with SO‐OCB. Given that training, managerial support, and autonomy associated with IM [88] may shape the relationship between CW and SO‐OCB, we further explored whether the IM × CW interaction was associated with SO‐OCB.

**TABLE 8:** Mediation effect testing results.

Path	Direct effect	Indirect effect	Bias‐corrected (95%)		*p*	Conclusion
			Lower bounds	Upper bounds		
IM ⟶ SAEL ⟶ SO‐OCB	0.089(^∗∗∗^)	0.029	0.019	0.045	< 0.001	Partial mediation
IM ⟶ DAEL ⟶ SO‐OCB	0.089(^∗∗∗^)	0.051	0.029	0.085	< 0.001	Partial mediation
IM ⟶ CW ⟶ SO‐OCB	0.089(^∗∗∗^)	0.004	−0.003	0.01	0.208	Not significant

Abbreviations: CW, care‐giving workload; DAEL, deep acting emotional labor; IM, internal marketing; SAEL, surface acting emotional labor; SO‐OCB, service‐oriented organizational citizenship behavior.

^∗^ *p* < 0.05.

^∗∗^ *p* < 0.01.

^∗∗∗^ *p* < 0.001.

### 4.8. Further Analysis

According to COR theory, the degree of fit between CW as a resource demand and IM as a resource supply could determine employees’ ability to balance stress and service performance. When well matched (e.g., high CW paired with strong support), resource depletion is offset, enabling stable SO‐OCB. In contrast, mismatches (e.g., high CW with low support) can create resource deficits and trigger withdrawal behaviors [89]. SDT further suggests that alignment between IM and CW enhances perceived organizational responsiveness. For example, when high CW is met with adequate empowerment, employees experience stronger intrinsic motivation, sustaining SO‐OCB. Conversely, unmet needs diminish motivation and weaken proactive service behaviors [90].

Prior research suggests that IM strategies—such as valuing employees and providing organizational support—can reduce stress [91], enhance job satisfaction [92], and foster organizational citizenship behavior by reducing role ambiguity and increasing commitment [93]. In eldercare, Chou and Robert [94] found that alignment between IM and CW leads to more stable service behaviors. However, excessive IM may also cause role ambiguity, weakening motivation and reducing informal behaviors like SO‐OCB. Edwards and Parry [95] further demonstrated that the fit between resource supply and demand exerts a nonlinear effect on employee outcomes—high fit yields stable results, while misfit leads to sharp declines.

Based on the above analysis, we hypothesize that the interaction between IM and CW has a nonlinear effect on SO‐OCB—a mechanism that warrants further investigation. To examine this dynamic, the present study employs hierarchical regression and RSM, offering refined theoretical insights and practical guidance for improving SO‐OCB in eldercare settings.

#### 4.8.1. Hierarchical Regression Analysis and RSM

We used hierarchical regression to examine the nonlinear and interactive associations of IM and CW with SO‐OCB. To aid interpretation of the regression coefficients, both focal predictors were centered at the midpoint of the five‐point scale: 3 was subtracted from the IM and CW scale scores before their squared and interaction terms were constructed. Control variables were entered in Step 1 (F1), followed by IM and CW in Step 2 (F2). The squared terms for IM and CW and their interaction term were entered in Step 3 (F3). A significant increase in the model’s explanatory power or a statistically significant coefficient for a higher‐order term prompted further examination of the corresponding nonlinear or interaction pattern.

When such effects were present, RSM was applied to visualize the interaction. A three‐dimensional plot was generated to illustrate how varying levels of congruence and incongruence between IM and CW influence SO‐OCB. As shown in Figure 3, the surface along the line of congruence (IM = CW) captures changes under matched conditions, while the line of incongruence (IM = –CW) reflects outcomes under mismatch. The shape and curvature of the surface provide the basis for testing the hypothesized interaction pattern.

**FIGURE 3:**
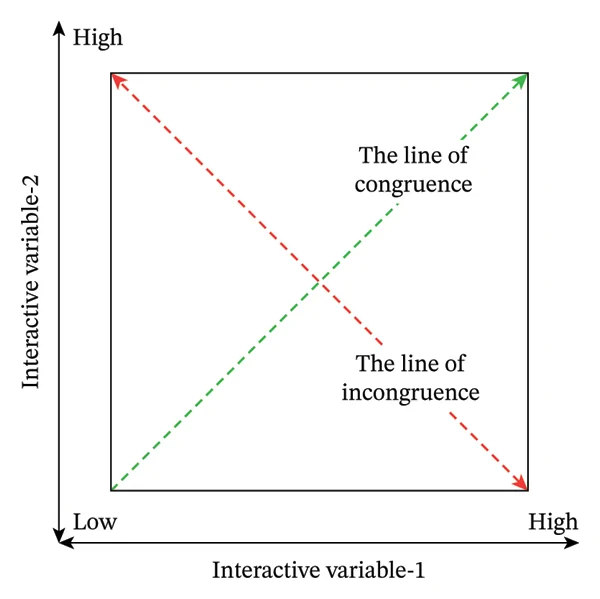
RSM diagram: lines of congruence and incongruence.

Response surface parameters were estimated following the procedure of Edwards and Parry [95]. Composite terms were first calculated as:

(1) x1=b1+b2,x2=b3+b4+b5.

Significance tests on *x*_1_ and *x*_2_ were then conducted. A significant positive *x*_1_ suggests that a high–high match between IM and CW yields more favorable outcomes than a low–low match, while a significant negative *x*_1_ indicates the opposite. A significant *x*_2_ reflects curvature along the line of congruence, indicating that outcomes vary with the level of matched values.

To examine the line of incongruence, two additional terms were calculated:

(2) y1=b1−b2,y2=b3+b4−b5.

A significant *y*_1_ indicates a linear trend under mismatched conditions, whereas a significant *y*_2_ suggests curvature along the incongruence line.

#### 4.8.2. Interaction Analysis Between IM and CW

Table 9 presents the response surface regression results. At least one quadratic term was statistically significant, providing evidence of a nonlinear association between IM, CW, and SO‐OCB. In Model F3, neither first‐order composite term was statistically significant: the slope along the line of congruence was *x*_1_ = *b*_1_ + *b*_2_ = 0.368 (SE = 0.308, 95% CI [−0.237, 0.972], *p* > 0.05), and the slope along the line of incongruence was *y*_1_ = *b*_1_ − *b*_2_ = 0.066 (SE = 0.217, 95% CI [−0.361, 0.492], *p* > 0.05). Thus, the first‐order slope was not statistically significant along either line.

**TABLE 9:** Hierarchical regression analysis results.

Variable	SO‐OCB		
	F1	F2	F3
Constant	3.969^∗∗∗^	4.079^∗∗∗^	3.632^∗∗∗^

Gender	−0.017	−0.077	−0.079

Age	0.057	0.022	0.022

Education	0.058	0.043	0.051

Marital status	−0.133	−0.051	−0.060

IM		0.199^∗∗∗^	0.217
CW		−0.193^∗∗∗^	0.151

IM^2^			−0.009

CW^2^			−0.061^∗^

IM ^∗^ CW			0.009

*R* ^2^	0.005	0.188	0.195

*F*	0.603	19.545^∗∗∗^	13.626^∗∗∗^
Adjusted *R* ^2^	−0.003	0.178	0.181

IM = CW (congruence line)	*x*_1_ = *b*_1_ + *b*_2_ = 0.368		
	*x*_2_ = *b*_3_ + *b*_4_ + *b*_5_ = −0.061^∗^		
IM = −CW (incongruence line)	*y*_1_ = *b*_1_ − *b*_2_ = 0.066		
	*y*_2_ = *b*_3_ + *b*_4_ − *b*_5_ = −0.079^∗^		

*Note: N* = 515. *b*_1_ = coefficient of IM; *b*_2_ = coefficient of CW; *b*_3_ = coefficient of IM^2^; *b*_4_ = coefficient of CW^2^; *b*_5_ = coefficient of IM × CW.

Abbreviations: CW, care‐giving workload; IM, internal marketing; SO‐OCB, service‐oriented organizational citizenship behavior.

^∗^ *p* < 0.05.

^∗∗∗^ *p* < 0.001.

By contrast, the CW^2^ term and the overall model containing the quadratic terms were statistically significant. Curvature along the line of congruence was also significant (*x*_2_ = *b*_3_ + *b*_4_ + *b*_5_ = −0.061, SE = 0.028, 95% CI [−0.117, −0.006], *p* < 0.05), consistent with an inverted U‐shaped pattern in SO‐OCB when IM and CW were at equal levels. As illustrated in Figure 4, SO‐OCB appeared to rise and then fall as IM and CW increased together from lower matched levels. This pattern suggests that the joint association of perceived IM support and care demands with SO‐OCB may vary across their levels.

**FIGURE 4:**
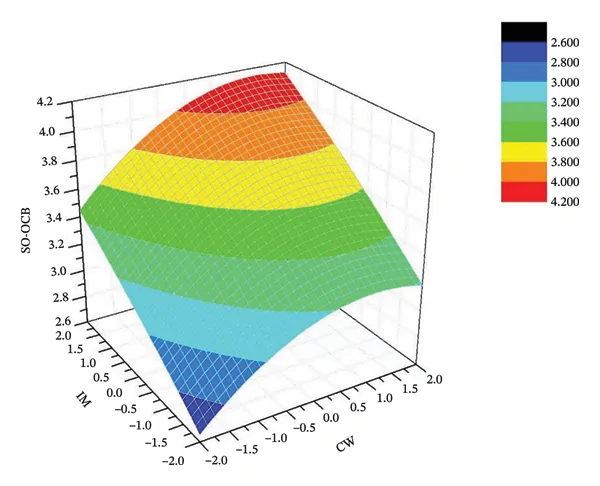
Response surface plot of IM and CW on SO‐OCB.

When IM and CW were incongruent, the quadratic term along the line of incongruence was significant (*y*_2_ = *b*_3_ + *b*_4_ − *b*_5_ = −0.079, SE = 0.045, 95% CI [−0.168, −0.003], *p* < 0.05), suggesting an inverted U‐shaped pattern in SO‐OCB along this line. In the response surface plot, SO‐OCB appeared to rise and then fall as the relative levels of IM and CW changed. This pattern may suggest that extra‐role service behavior is lower when the two differ substantially, whether IM or CW is relatively higher. The surface describes associations within this sample; longitudinal research in independent samples, drawing on different sources of care‐work and employee‐behavior data, is needed to examine this pattern further.

## 5. Conclusions and Implications

### 5.1. Key Findings

Using survey data from 515 respondents, this study examined the relationships linking IM to SO‐OCB among medical and nursing staff in nursing homes. Three findings emerged: (1) IM was positively associated with SO‐OCB. (2) The results were consistent with partial mediation through EL. SAEL was negatively associated with SO‐OCB, whereas DAEL was positively associated with it. (3) CW was negatively associated with SO‐OCB, but the proposed indirect path through CW was not supported. We also conducted an exploratory response surface analysis to examine the joint association of IM and CW with SO‐OCB. Along the line of congruence (IM = CW), the fitted surface showed an inverted U‐shaped trend in predicted SO‐OCB as IM and CW increased together from low levels. This pattern suggests a possible nonlinear association between their shared level and SO‐OCB.

### 5.2. Theoretical Contributions

This study’s theoretical contributions are mainly reflected in three areas:

1. This study extends research on the relationship between IM and employee behavior to the nursing home setting, adding context‐specific evidence on the link between employee‐oriented management practices and SO‐OCB. Previous studies have examined the effects of IM on employee satisfaction [6] and job performance [96], or investigated SO‐OCB in manufacturing and educational services [97, 98]. Eldercare involves continuous interactions and substantial differences in individual needs. Alongside their assigned tasks, staff must respond to older adults’ needs that cannot be fully met through standardized procedures [99]. By examining the relationship between IM and SO‐OCB among eldercare staff in this setting, the study provides empirical evidence for understanding the role of employee‐oriented management practices in ongoing care relationships.
2. This study adds EL to the explanation of the relationship between IM and SO‐OCB and refines that explanation by distinguishing SAEL from DAEL. Previous research has linked EL to emotional exhaustion and customer‐oriented behavior [46, 100]. Our findings suggest that how employees manage emotional demands may be an important part of the pathway linking IM practices with SO‐OCB. Examining SAEL and DAEL separately brings into view differences that an overall measure of EL could obscure. It also offers a more precise account of how distinct emotion regulation strategies relate to the observed indirect associations in eldercare.
3. This study also provides exploratory evidence on the joint association of IM and CW with SO‐OCB from a COR perspective. Previous research has examined SO‐OCB in relation to psychological empowerment [101], leader–member exchange [102], job stress [103], and workload [104]. We consider both the supportive resources represented by IM and the sustained effort required by care work, extending attention beyond either factor alone to the levels at which they occur together. The nonlinear trend in the response surface suggests that SO‐OCB may not continue to rise as support and workload increase together. This points to the value of considering task demands when interpreting the association between organizational resources and SO‐OCB. It also provides a basis for examining different combinations of support and workload in future research and poses a more specific question for applying COR in eldercare settings.

### 5.3. Practical Implications

First, nursing managers should tailor support to staff needs and establish structured development plans. The positive association between IM and SO‐OCB suggests the value of translating IM into specific forms of staff support and development. Through regular conversations and assessments of role‐specific competencies, nursing supervisors can identify needs related to care skills, decision‐making authority, and coordination across roles, then plan development in stages. New staff, for example, could receive on‐the‐job mentoring; staff seeking to strengthen particular care skills could undertake scenario‐based training; and experienced staff could be offered advanced learning and mentoring opportunities. Each stage should have clear learning objectives, a designated person responsible, and a scheduled feedback point. Managers can assess progress by observing how staff apply their skills in practice. Concerns about authority or procedures should also inform changes to role responsibilities, delegated authority, and coordination arrangements.

Second, emotional support should be part of routine care management. The differing associations of SAEL and DAEL with SO‐OCB suggest that managers should attend to the ways staff handle emotional demands and the difficulties they face. Nursing supervisors could hold regular discussions of challenging care situations, allowing staff to share experiences of communicating with residents and families. Peer support, one‐to‐one conversations with supervisors, and referral routes for professional consultation would provide additional ways to seek help. Where staff repeatedly undertake emotionally demanding tasks, supervisors could consider task rotation and recovery time alongside continuity of care and staffing needs. Training supervisors to recognize emotional distress, listen, and respond supportively may further strengthen this approach.

Third, nursing homes should monitor workload alongside staffing levels. CW was negatively associated with SO‐OCB, but the proposed indirect path through CW was not supported; these findings are insufficient to establish IM as a means of reducing workload. Nursing supervisors could review residents’ care needs, staff actually on duty, task interruptions, and overtime by shift, together with staff feedback. This would help identify periods of concentrated demand and staffing gaps and inform adjustments to schedules, skill mix, and task allocation. Persistent mismatches between task volume and available staff warrant assessment of additional staffing or changes to work processes. Scheduling should also account for the time needed for training, mentoring, and emotional support. Managers can adjust the content and timing of support to suit different roles and shifts and assess its implementation through staff feedback, changes in workload, and service performance.

### 5.4. Limitations and Future Research Directions

This study has several limitations. First, the main variables were self‐reported and may be subject to shared response tendencies, social desirability, and recall bias. CW captures perceived rather than objectively recorded workload. Without objective indicators, we could not fully assess how perceived workload relates to actual task demands. Future studies could combine employee reports with supervisor or colleague ratings and records of care tasks, working hours, and staffing. Second, the intervals between the three survey waves were relatively short and may not capture longer‐term changes. Separating measurements in time is also insufficient to establish causality. Longer follow‐up periods, repeated measurements across more waves, and intervention or quasi‐experimental designs could help examine the proposed processes. Third, employees within the same nursing home share management arrangements and a work environment, so their responses may be correlated. This study did not fully account for clustering within institutions, which may affect standard error estimates and statistical significance. Future analyses could use suitable multilevel models or cluster‐robust methods, taking the number of institutions and the sample structure into account. Finally, all participating nursing homes were in Beijing. The applicability of the findings to other regions and types of institutions remains uncertain. Broader sampling and replication of the mediation and response surface findings in independent samples are needed.

## Author Contributions

An‐Jin Shie: conceptualization, methodology, funding acquisition, project administration, writing–original draft, and writing–review and editing; Junhui Liu: conceptualization, methodology, data curation, investigation, writing–original draft, and writing–review and editing; Jingxian Xie: data curation, investigation, methodology, and writing–review and editing; En‐Min Xu: conceptualization, formal analysis, validation, visualization, writing–original draft, and writing–review and editing.

## Funding

This research was supported by the Research Projects on Basic Theoretical Studies of Philosophy and Social Sciences Guided by Marxism in Fujian Universities (Key Project) (Grant Number: FJ2025MGCA043) and the Quanzhou Social Science Planning Project (Key Project) (Grant Number: 2024C03).

## Ethics Statement

All of the investigated data were approved by the Institutional Review Board of Huaqiao University (IRB No: H‐2024‐1006).

## Consent

Informed consent was obtained from all medical and nursing staff who participated in the questionnaire survey for this study.

## Conflicts of Interest

The authors declare no conflicts of interest.

## Supporting Information

Additional supporting information can be found online in the Supporting Information section.

## Supporting information


**Supporting Information** Supporting Material 1. Questionnaire on internal marketing and service‐oriented organizational citizenship behavior among medical and nursing staff in nursing homes. This file provides the questionnaire used in this study, including the measurement items for the study variables.

## Data Availability

The data that support the findings of this study are available from the corresponding author, upon reasonable request.

## Appendix A

**Table A1:** List of abbreviations used in the article.

Abbreviation	Full term
AVE	Average variance extracted
CFA	Confirmatory factor analysis
CITC	Corrected item–total correlation
CMB	Common method bias
COR	Conservation of resources
CR	Composite reliability
CW	Care‐giving workload
DAEL	Deep acting emotional labor
EL	Emotional labor
IM	Internal marketing
RSM	Response surface methodology
SAEL	Surface acting emotional labor
SDT	Self‐determination theory
SEM	Structural equation modeling
SO‐OCB	Service‐oriented organizational citizenship behavior
