# Supplementary material for: Internal Marketing and Service‐Oriented Organizational Citizenship Behavior Among Medical and Nursing Staff in Nursing Homes
Source: J Nurs Manag. 2026 Nov 15;2026:1415183. doi: 10.1155/jonm/1415183 (PMC13770269; doi:10.1155/jonm/1415183)
Supplement: Supplementary file 1 [file JONM-2026-1415183-s001.docx]

**Questionnaire on internal marketing and service-oriented organizational citizenship behavior among medical and nursing staff in nursing homes**

| Dear Participant，  Greetings! First of all, we sincerely thank you for taking the time to complete this questionnaire. This is an academic survey designed to explore issues related to how internal marketing in elderly care institutions influences the service-oriented organizational citizenship behavior of care staff. The target respondents are medical and nursing staff working in nursing homes. Please be assured that the information you provide will be used solely for academic research purposes and will be kept strictly confidential. | | | | | | | | | |
| --- | --- | --- | --- | --- | --- | --- | --- | --- | --- |
| *The following section collects basic demographic information. All responses are used solely for aggregate statistical analysis and will remain strictly confidential. Please feel assured when completing this section. | | | | | | | | | |
| 1 Are you a medical or nursing staff member in a nursing home?  ☐ Yes     ☐ No | | | | | | | | | |
| 2 Gender:  ☐ Male    ☐ Female | | | | | | | | | |
| 3 Age:  ☐ 25 or below  ☐ 26–30 years old  ☐ 31–35 years old  ☐ 36–40 years old  ☐ 41 or above | | | | | | | | | |
| 4 Education level:   ☐ High school or below   ☐ Junior college (Associate degree)   ☐ Bachelor’s degree   ☐ Master’s degree or above | | | | | | | | | |
| 5 Marital status:   ☐ Single    ☐ Married | | | | | | | | | |
|  |  | | |  |  |  |  |  |  |
| **[Section 1]** The following questions are mainly about your evaluation of internal marketing practices in your nursing home. They reflect how the institution treats its employees and how you personally perceive these aspects in your daily work. Please select the option that best represents your actual feelings and experiences **(single choice per item)**. A higher number indicates that the statement more accurately reflects the reality. | | | Strongly Disagree | | Disagree | Neutral | Agree | Strongly Agree |  |
|  |  |  | 1 | | 2 | 3 | 4 | 5 |  |
| 1 | | The nursing home’s work regulations clearly define my care-giving responsibilities. | ☐ | | ☐ | ☐ | ☐ | ☐ |  |
| 2 | | My supervisor provides concrete support and empowerment in my work. | ☐ | | ☐ | ☐ | ☐ | ☐ |  |
| 3 | | The flat management structure of the nursing home motivates me to work more proactively. | ☐ | | ☐ | ☐ | ☐ | ☐ |  |
| 4 | | I am authorized to handle tasks flexibly based on residents’ needs. | ☐ | | ☐ | ☐ | ☐ | ☐ |  |
| 5 | | My medical and nursing work is recognized by both supervisors and residents. | ☐ | | ☐ | ☐ | ☐ | ☐ |  |
| 6 | | The nursing home communicates future plans and goals to employees. | ☐ | | ☐ | ☐ | ☐ | ☐ |  |
| 7 | | A suggestion box is available in the nursing home for employees to express opinions. | ☐ | | ☐ | ☐ | ☐ | ☐ |  |
| 8 | | I am able to independently go above and beyond my job duties. | ☐ | | ☐ | ☐ | ☐ | ☐ |  |
| 9 | | I have partial decision-making power and the ability to act on the nursing home’s goals and plans. | ☐ | | ☐ | ☐ | ☐ | ☐ |  |
| 10 | | I like the corporate culture of our nursing home. | ☐ | | ☐ | ☐ | ☐ | ☐ |  |
| 11 | | The nursing home provides me with various training opportunities. | ☐ | | ☐ | ☐ | ☐ | ☐ |  |
| 12 | | The training programs offered by the nursing home meet my actual needs. | ☐ | | ☐ | ☐ | ☐ | ☐ |  |
| 13 | | The nursing home encourages me to participate in training that benefits my career development. | ☐ | | ☐ | ☐ | ☐ | ☐ |  |
| 14 | | Senior managers in the nursing home regularly show concern for my emotional well-being. | ☐ | | ☐ | ☐ | ☐ | ☐ |  |
| 15 | | Leadership encourages and supports me to demonstrate my talents. | ☐ | | ☐ | ☐ | ☐ | ☐ |  |
| 16 | | The training sessions and activities help me understand current development policies. | ☐ | | ☐ | ☐ | ☐ | ☐ |  |
| 17 | | The nursing home always provides fair competition opportunities. | ☐ | | ☐ | ☐ | ☐ | ☐ |  |
| 18 | | Leadership is willing to adopt development suggestions proposed by regular staff. | ☐ | | ☐ | ☐ | ☐ | ☐ |  |
| 19 | | The nursing home values and responds to employees’ reasonable needs. | ☐ | | ☐ | ☐ | ☐ | ☐ |  |
| 20 | | Working in the nursing home makes me feel valued. | ☐ | | ☐ | ☐ | ☐ | ☐ |  |
| 21 | | The benefits and promotion opportunities in the nursing home are clearly defined. | ☐ | | ☐ | ☐ | ☐ | ☐ |  |
| 22 | | The nursing home offers support for employees’ families, such as counseling services. | ☐ | | ☐ | ☐ | ☐ | ☐ |  |

| **[Section 2]** The following items are intended to assess your perception of care-giving workload and reflect how intense your work feels while working in the nursing home. Please select the option that best represents your actual experience **(single choice per item)**. A higher number indicates that the statement more accurately reflects the reality. | | | Strongly Disagree | | Disagree | Neutral | Agree | Strongly Agree |
| --- | --- | --- | --- | --- | --- | --- | --- | --- |
|  |  |  | 1 | | 2 | 3 | 4 | 5 |
| 1 | | I feel stressed when the nursing home requires me to complete tasks quickly. | ☐ | | ☐ | ☐ | ☐ | ☐ |
| 2 | | I strongly resist being pushed to put in my maximum effort at work. | ☐ | | ☐ | ☐ | ☐ | ☐ |
| 3 | | When assigned tasks beyond my job responsibilities, I feel wronged and consider quitting. | ☐ | | ☐ | ☐ | ☐ | ☐ |
| 4 | | Under intense work pressure, I am unable to complete tasks on time. | ☐ | | ☐ | ☐ | ☐ | ☐ |
| 5 | | Excessive workload affects me physically and mentally, leaving me exhausted. | ☐ | | ☐ | ☐ | ☐ | ☐ |
| 6 | | Providing care to elderly residents requires full concentration, which makes me extremely fatigued. | ☐ | | ☐ | ☐ | ☐ | ☐ |
| 7 | | Even when I devote myself to caring for the elderly, I am still blamed for poor work attitude. | ☐ | | ☐ | ☐ | ☐ | ☐ |
| 8 | | The nursing home sets very high expectations for my work performance, which causes me significant stress. | ☐ | | ☐ | ☐ | ☐ | ☐ |
| 9 | | The physical condition of the residents makes care-giving feel risky and stressful. | ☐ | | ☐ | ☐ | ☐ | ☐ |
| 10 | | When I fail to do my job well, I feel guilty toward the nursing home. | ☐ | | ☐ | ☐ | ☐ | ☐ |
|  |  | | |  |  |  |  |  |
| **[Section 3]** The following items are designed to assess your emotional experiences at work and how you manage emotions while working in the nursing home. Please select the option that best represents your actual feelings **(single choice per item)**. A higher number indicates that the statement more accurately reflects the reality. | | | Strongly Disagree | | Disagree | Neutral | Agree | Strongly Agree |
|  |  |  | 1 | | 2 | 3 | 4 | 5 |
| 1 | | I pretend to be in a good mood when interacting with elderly residents, even when I am actually unhappy inside. | ☐ | | ☐ | ☐ | ☐ | ☐ |
| 2 | | My behavior and attitude when caring for elderly residents feel like acting. | ☐ | | ☐ | ☐ | ☐ | ☐ |
| 3 | | The emotions I display in care-giving are faked to meet job requirements, or I might lose bonuses. | ☐ | | ☐ | ☐ | ☐ | ☐ |
| 4 | | I would rather fake the emotions expected in care-giving than change how I truly feel. | ☐ | | ☐ | ☐ | ☐ | ☐ |
| 5 | | I genuinely feel happy when interacting with elderly residents. | ☐ | | ☐ | ☐ | ☐ | ☐ |
| 6 | | While working, I treat elderly residents like my own family and serve them wholeheartedly. | ☐ | | ☐ | ☐ | ☐ | ☐ |
| 7 | | Even when elderly residents are unreasonable, I try to understand their perspective and solve the problem. | ☐ | | ☐ | ☐ | ☐ | ☐ |
| 8 | | I truly enjoy working in this field and strive to complete every task with dedication. | ☐ | | ☐ | ☐ | ☐ | ☐ |
|  |  | | |  |  |  |  |  |
| **[Section 4]** The following items are intended to assess your perceptions of service-oriented organizational citizenship behavior. They reflect your attitudes and proactive behaviors toward the nursing home in the course of your work. Please select the option that best represents your actual experience **(single choice per item)**. A higher number indicates that the statement more accurately reflects the reality. | | | Strongly Disagree | | Disagree | Neutral | Agree | Strongly Agree |
|  |  |  | 1 | | 2 | 3 | 4 | 5 |
| 1 | | I tell others that my work environment is good. | ☐ | | ☐ | ☐ | ☐ | ☐ |
| 2 | | I actively share the strengths of my nursing home with others. | ☐ | | ☐ | ☐ | ☐ | ☐ |
| 3 | | I take the initiative to do things that benefit the nursing home. | ☐ | | ☐ | ☐ | ☐ | ☐ |
| 4 | | I encourage friends and their elderly family members to consider living in the nursing home. | ☐ | | ☐ | ☐ | ☐ | ☐ |
| 5 | | I take the initiative to introduce or promote the services of my nursing home. | ☐ | | ☐ | ☐ | ☐ | ☐ |
| 6 | | I strictly follow the nursing home’s rules and regulations when serving elderly residents. | ☐ | | ☐ | ☐ | ☐ | ☐ |
| 7 | | I respond promptly to the needs and concerns of elderly residents. | ☐ | | ☐ | ☐ | ☐ | ☐ |
| 8 | | I rarely make mistakes in my care-giving work at the nursing home. | ☐ | | ☐ | ☐ | ☐ | ☐ |
| 9 | | I have always enjoyed my current care-giving work in the nursing home. | ☐ | | ☐ | ☐ | ☐ | ☐ |
| 10 | | I remain calm and polite to elderly residents under all emotional conditions. | ☐ | | ☐ | ☐ | ☐ | ☐ |
| 11 | | I encourage my colleagues to suggest ways to improve care-giving service attitudes in the nursing home. | ☐ | | ☐ | ☐ | ☐ | ☐ |
| 12 | | I propose constructive ideas related to healthcare improvements for the nursing home. | ☐ | | ☐ | ☐ | ☐ | ☐ |
| 13 | | I frequently share solutions to difficult care-giving problems with my colleagues. | ☐ | | ☐ | ☐ | ☐ | ☐ |
| 14 | | I bring care-giving-related documents home for further study. | ☐ | | ☐ | ☐ | ☐ | ☐ |
| 15 | | I actively participate in all activities organized by the nursing home. | ☐ | | ☐ | ☐ | ☐ | ☐ |

This concludes the questionnaire.

Please kindly check if any items have been left unanswered.

We sincerely appreciate your participation and support.

Wishing you good health and all the best.
